# Influence of electric potential-induced by atmospheric pressure plasma on cell response

**DOI:** 10.1038/s41598-023-42976-4

**Published:** 2023-09-25

**Authors:** Takamasa Okumura, Chia-Hsing Chang, Kazunori Koga, Masaharu Shiratani, Takehiko Sato

**Affiliations:** 1 Faculty of Information Science and Electrical Engineering, Kyushu University, Fukuoka, Fukuoka 819-0395 Japan; 2 Institute of Fluid Science, Tohoku University, Sendai, Miyagi 980-8577 Japan

**Keywords:** Plasma physics, Electrical and electronic engineering

## Abstract

Plasma irradiation leads not only active species, but also reactive chemical species, ultraviolet light, electric fields, magnetic fields, and shock waves. To date the effects of reactive chemical species have been mainly discussed. To understand the biological effect caused by an electric potential induced with an atmospheric-pressure plasma, the behavior of cell stimulated by electric potential was investigated using HeLa cell. The cell concentration assay revealed that less than 20% of cells inactivated by potential stimulation and the remained cells proliferate afterward. Fluorescent microscopic observation revealed that potential stimulation is appreciable to transport the molecules through membrane. These results show that potential stimulation induces intracellular and extracellular molecular transport, while the stimulation has a low lethal effect. A possible mechanism for this molecular transport by potential stimulation was also shown using numerical simulation based on an equivalent circuit of the experimental system including adhered HeLa cell. The potential formation caused by plasma generation is decisive in the contribution of plasma science to molecular biology and the elucidation of the mechanism underlying a biological response induction by plasma irradiation.

## Introduction

Recently, atmospheric-pressure plasma has been intensively investigated for biomedical^1–4^, such as the inactivation of bacteria including antibiotic-resist germs, fungi, spores or viruses and the hemostasis^5–8^, and agricultural applications^9–12^, such as germination enhancement, subsequent growth improvement and DNA methylation alterations of plant seeds^13–15^. Hence, the plasma irradiation can be applied in medical and agricultural treatments. During the plasma irradiation, targets are covered with liquid phase and thus it is necessary to investigate the behavior of cells in liquid by plasma irradiation^16^.

**Figure 1:**
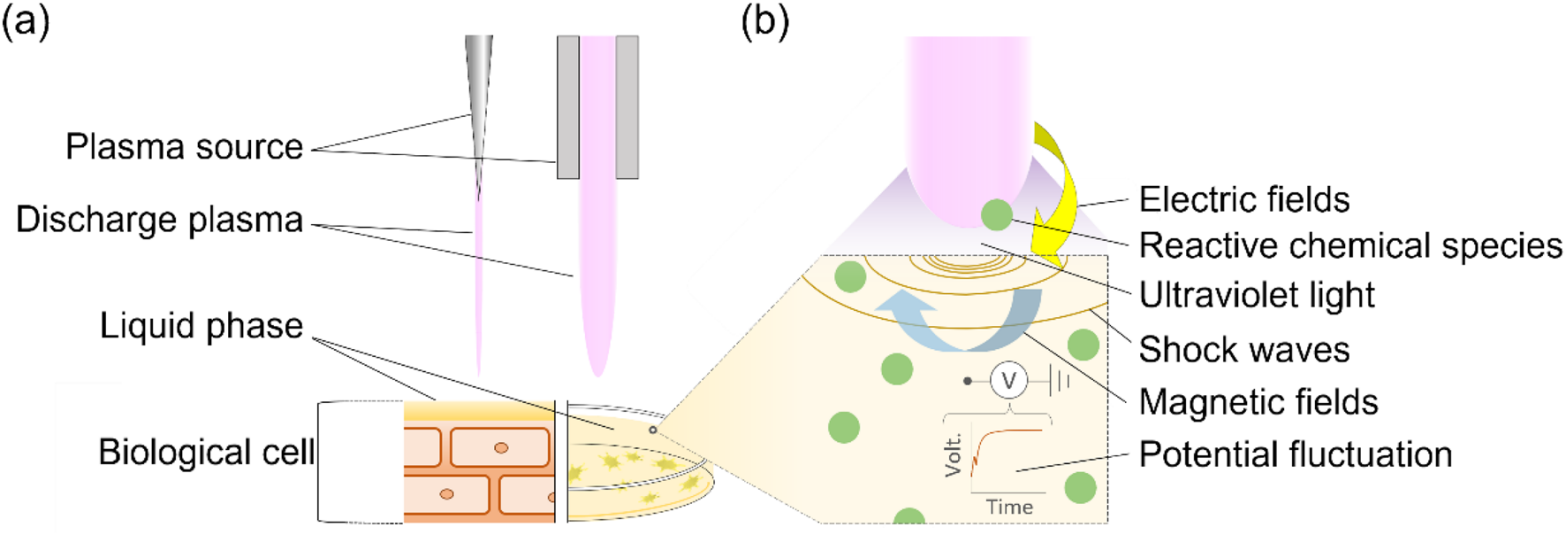
Plasma irradiation model for cells/tissues/organs.

A single biological effect of plasma-induced physical and chemical elements remains an important issue^17^. Although the plasma acts as a source of several active agents, *e.g.*, reactive chemical species, charged particles, ultraviolet light, electric fields, magnetic fields, and shock waves (Fig. 1) and one of these or combination induces a biological response^17–19^, the mechanism underlying the effects caused by the plasma has been discussed mainly in terms of reactive species to date. Recently, Chang et al*.* and his colleagues constructed the system to evaluate the effect of plasma-induced nanosecond pulsed current and chemical factors on human HT-1080 cells activities and observed a significant increase in cell migration along with altered cell morphology^19–22^. Mendis et al*.* theoretically discussed the mechanism underlying physical disruption of cell membranes due to plasma irradiation in terms of potential^23^. Furthermore, Yano et al*.* showed that pulsed electric field application at a low frequency range less than 1 MHz affects cell activity depending on the frequency^8^. Considering that the accumulated charge also has a biological effect in and of themselves^8,23^, the plasma-induced electric potential on living tissues is also crucial for the biological effects.

To understand the biological effect of the electric potential of plasma, it is essential to exclude the influence of chemical species. We constructed a novel experimental system for cell stimulation that could physically isolate cells from reactive species generated at discharge region and thus allowed us to evaluate the direct effects of electrical potential fluctuation on cellular response. A possible mechanism for the cell response to potential stimulation was also shown using numerical simulation based on an equivalent circuit of the experimental system including adhered cell. Defining the interactions between the cell and the electric potential could help us understand the mechanisms complementary to biological effect induced by plasma irradiation. Our main goal was to investigate whether electrical potential affect cellular responses and provide the probable mechanism of the cell response.

## Results and discussion

### Discharge assessment assay of medium

**Figure 2:**
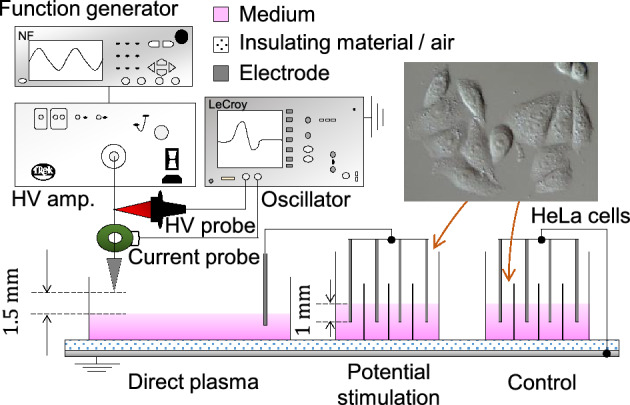
Experimental set-up.

**Figure 3:**
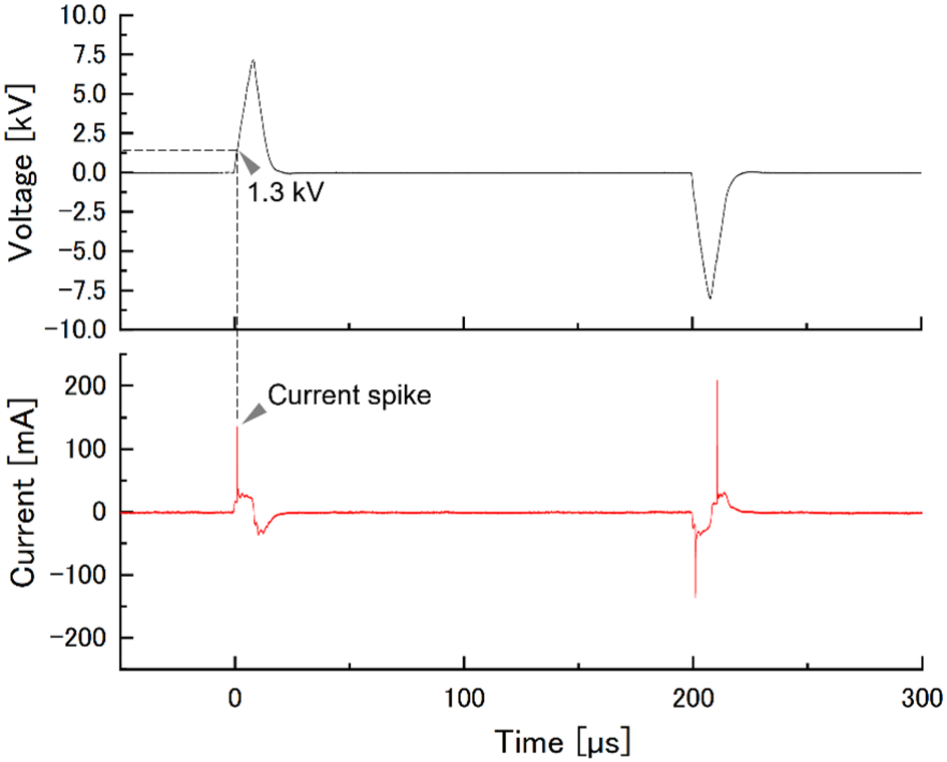
Typical waveforms of the applied voltage to a needle electrode and current flowing between the needle electrode and the ground.

**Figure 4:**
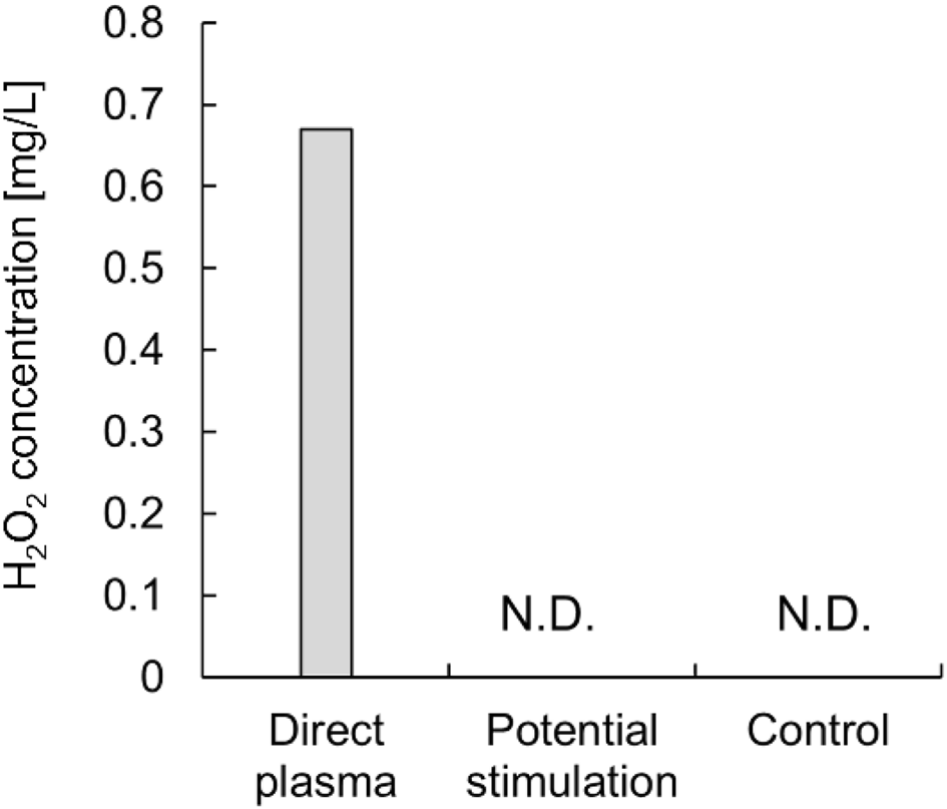
H_2_O_2_ concentration of each medium for direct plasma, potential stimulation, and control. The duration of plasma irradiation was 60 min. N.D. shows not detected with a detection limit of 0.05 mg/L. The error bar of direct plasma is invisible due to the standard deviation was zero.

HeLa cell suspension in a culture medium was prepared in wells of a 96-well plate and incubated for 24 h, the cells were adhered to the bottom surface and used for potential stimulation as shown in Fig. 2. The experimental parameters were listed in Table 1. We prepared cells in two conditions; one experienced exposure to potential stimulation and the other was placed in the grounded medium (control). A pulsed discharge that propagates between the needle and the medium forms the potential in the liquid^24,25^. The potential fluctuation of the medium at the direct discharges was transferred through a SUS 316 cylindrical wire to the medium including HeLa cells. To generate plasma on the medium of direct plasma a pulsed voltage was applied to the needle electrode. Figure 3 shows typical waveforms of the applied voltage to the needle electrode and current flowing through the needle and the ground. Current spikes, corresponding to discharge occurrence, appeared with voltage changes. The first discharge occurs at 1.3 kV. The pulse width of the discharge current spikes are 88 ns, indicating that once a discharge propagates to the other side then it stops. These results show that discharge mode did not shift to arcing from streamer. The typical streamer channel is < 30 Td with the electron temperature *T*_*e*_ < 2.7 eV^26^. Such electrons mainly vibrationally excite N_2_ and O_2_ in the air and generate NO in the atmosphere^27^. Therefore, the media with cell were closed to prevent contamination of chemical species. If a discharge is generated in the medium with cell, the effect of potential stimulation on the cells cannot be extracted. When an electric discharge occurs, H_2_O_2_, relatively long-lived RONS, is generated in the medium by the chemical reaction through R1 and R2^28^.

$$ {\text{H}}_{{2}} {\text{O}} + P \to^{*} {\text{OH}} +^{*} {\text{H}} + P\quad \left( {{\text{R}}1} \right) $$

$$^{*} {\text{OH}} +^{*} {\text{OH}} + {\text{M}} \to {\text{H}}_{{2}} {\text{O}}_{{2}} + {\text{M}}\quad \left( {{\text{R}}2} \right) $$

where *P* is an energetic particle from the plasma (e.g., electron) and M is a collision partner (e.g., N_2_ or O_2_). Figure 4 shows H_2_O_2_ concentration in the medium below the needle electrode (direct plasma), that of potential stimulation, and that of control after 60 min-plasma generation. H_2_O_2_ concentration shows 0.67 ± 0.00 mg/L for direct plasma (n = 3). In contrast, H_2_O_2_ was not detected in the medium with and without potential stimulation. When only one electrode with a certain potential is in contact with a liquid, the potential of the liquid is equipotential with the electrode and thus no discharge involving a chemical reaction occurs theoretically. This is consistent with the result in Fig. 4. On the other hand, if consider hypothetically that the electrode immersed in the liquid has a high potential and plasma is generated, the discharge should be localized between the tip of the electrode and the liquid due to the concentration of the electric field^29^. In such early phenomenon, electrons use the region created by vaporization of liquid due to Joule heating to accelerate^29^. Note that in reference 29 the high voltage electrode and the ground electrode were placed in a liquid at a short distance. The region would be dominated by H_2_O and the chemical species produced in medium including cells would be dominated by *OH and hydrogen atom if the discharge occurs^29,30^ Sato et al*.* also indicated that second positive and first negative band emissions of nitrogen were not detected in the region^29^. From the above, potential stimulation on HeLa cells will be discussed, excluding the effect of RONS. Additionally, H_2_O_2_ is a chemical species that is a major contributor to the stress that cells undergo during plasma irradiation^31^. We preliminary found that nitrite HNO_2_ has no inactivation effect up to concentrations of 2.5 mM (118 mg/L) and nitrate HNO_3_ up to 5 mM (315 mg/L), H_2_O_2_ even below 150 µM inactivates HeLa cells. This indicates that HNO_2_ and HNO_3_, which are produced in large amounts by plasma irradiation in air gas to liquid, have relatively low toxicity to HeLa cells comparing to H_2_O_2_.

**Table 1:** Parameters of cell preparation and electric potential stimulation.

Cell suspension volume in a well	100 μL
Initial cell concentration	6.0 × 10^4^ or 1.0 × 10^5^cell/mL
Incubation condition of HeLa cell	37 °C with 5% Co_2_ for 24 h
Culture medium composition	Minimun essential medium with 10% fetal bovine serum and 2% pencilin–Streptomycin mixed solution
Distance between a needle tip and a medium’s surface	1.5 mm
Tip radius of needle electrode	40 μm
Pulsed voltage applied to the needle	Amplitude of ± 7.5 kV_pp_, a rise time of 8 μs, width of 9 μs, and pulse repetition rate of 5 kpps
Treatment time	30 or 60 min

### Cell concentration assay

**Figure 5:**
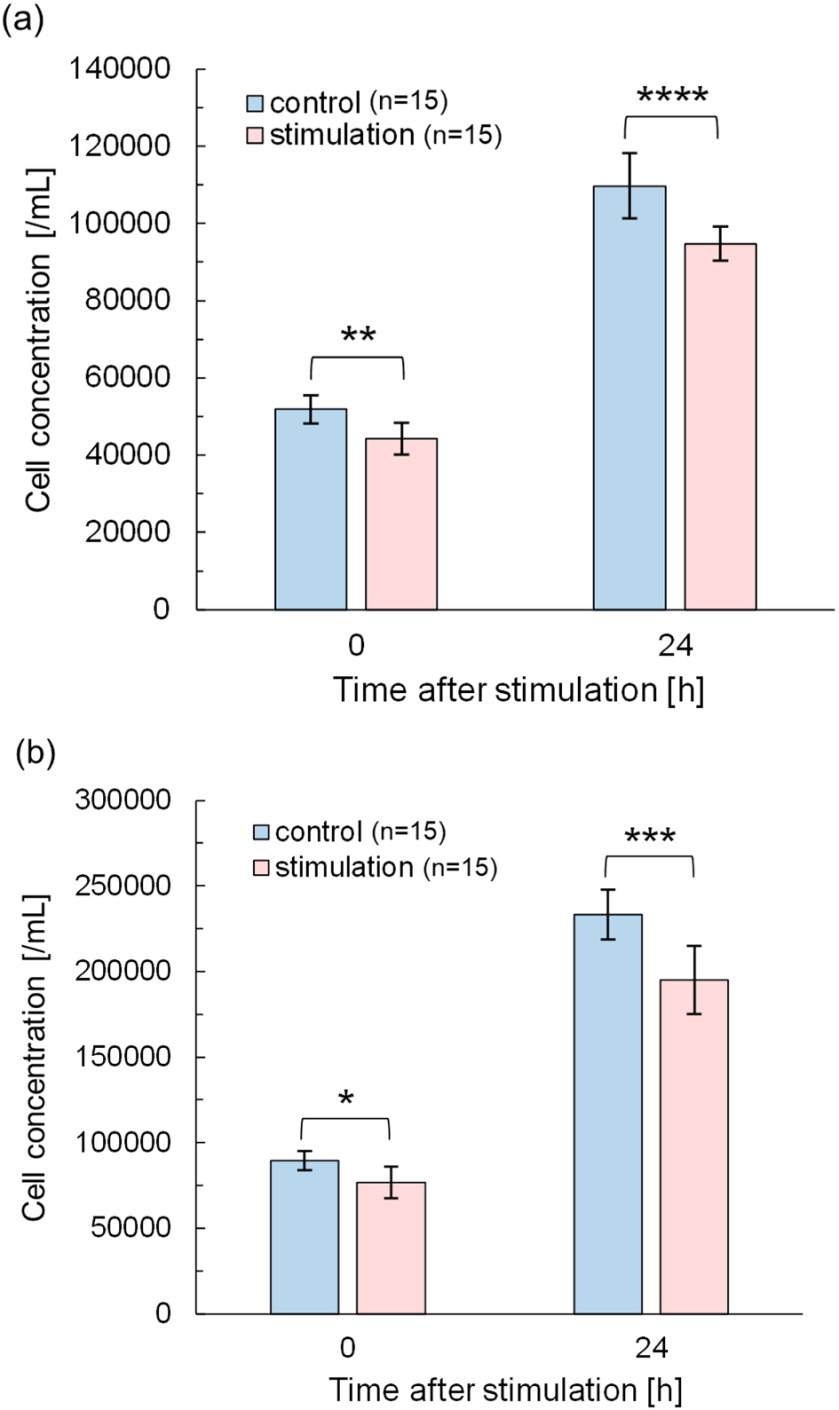
Cell concentration after a potential stimulation for 30 min (**a**) with and (**b**) without replacing medium with PBS. The time between stimulation treatment and measurement was 0 and 24 h. Initial cell concentration was 6.0 × 10^4^ cell/mL in (**a**) and 1.0 × 10^5^ cell/mL in (**b**). Statistical test of two tailed *t*-test was performed (*p < 0.05, **p < 0.01, ***p < 0.005, ****p < 0.001). %Decrease was 85% for 0 h and 86% for 24 h in (**a**) and 86% for 0 h and 84% for 24 h in (**b**).

The effect of potential stimulation on cells was evaluated by changes in the cell activity. Figure 5 shows changes in the concentration of living cells in the medium with and without potential stimulation for 30 min. The cell concentrations were measured after treatment (0 h) and after 24 h of incubation since the treatment (24 h). Figure 5a was obtained after immediately (within 1–2 min after plasma irradiation) replacing the medium with phosphate-buffered saline (PBS) followed by cell concentration assay. The concentration of treated cells decreases in both 0 h and 24 h. Most cells remain active at 0 h after the treatment unless there is a steep disruption of cell membranes due to direct plasma irradiation. Notice that cell concentration was obtained by adding the reagent to the sample and incubating for 4 h according to the product protocol. Therefore, the possible mechanism of the decrease in cell concentration even at 0 h is due to a rapid cell inactivation during potential stimulation and a gradual cell inactivation during 4 h of incubation for assay. We evaluated %decrease due to potential stimulation by Eq. (1).

1 $$\%Decrease=\frac{{n}_{\mathrm{s}} }{{n}_{\mathrm{c}}}\times 100 [\%]$$

where *n*_s_ is the concentration of living cell with potential stimulation (/mL) and *n*_c_ is that without potential stimulation (/mL). In Fig. 5., %decrease is 85% at 0 h (*p* = 0.0055) and 86% at 24 h (*p* = 0.00099), respectively. HeLa cells adheres on the bottom using proteins such as extracellular matrix and adhesion molecules^32^. Local electric fields created by potential fluctuations might affect their protein function by changing its conformation^33,34^. Additionally, a global feature of plasma irradiation is instantaneous cell detachment from the bottom. I.E. Kieft et al. observed the detachment from the bottom for Chinese hamster ovary cells, an epithelial cell line, within a few minutes after a plasma irradiation for 5 s and the reattachment at 1 h after plasma irradiation^35^. Since the cell concentration of Fig. 5a was obtained by replacing the medium with PBS immediately (within 1–2 min after plasma irradiation) after potential stimulation, the cells detached by potential stimulation might be discarded leading cell concentration decrease. Figure 5b shows the concentration of cells counted after stimulation without replacing the medium with PBS. Nevertheless, %decrease was 84% ​​at 0 h (*p* = 0.012) and 84% at 24 h (*p* = 0.0033). This result is in good agreement with the result in Fig. 5a. Consequently, potential stimulation affects the cells within 4 h at the longest after potential stimulation and these effects are not explained in terms of the detachment.

To discuss the subsequent effects, a proliferation ratio *R*_p_ was obtained by Eq. (2).

2 $${R}_{\mathrm{p}}=\frac{{n}_{24\mathrm{h}} }{{n}_{0\mathrm{h}}}$$

where *n*_24h_ is the concentration of cells incubated for 24 h after potential stimulation and *n*_0h_ is that without incubation after potential stimulation. *R*_p_ is 2.11 and 2.14 for control and stimulation in Fig. 5a, and 2.61 and 2.55 in Fig. 5b, respectively. Considering that the initial cell concentration was 6.0 × 10^4^ cell/mL in Fig. 5a and 1.0 × 10^5^ cell/mL in Fig. 5b, it is natural that Fig. 5a shows higher cell proliferation ratios. However, it should be noted that the ratio is maintained at the same level as the control group, even though potential stimulation reduces the concentration of living cells. The cells may recover from temporal damage due to the stimulation. Considering that the doubling time of HeLa cells is within 24 h^36,37^, the cells may recover in a few hours after the stimulation. Alternatively, potential stimulation may shorten the cell cycle. The mechanism underlying how the cell proliferation rate is compensated (Fig. 5) should be clarified in the future. In this study, further experiments were conducted on the effects of potential fluctuations during plasma generation on cell membranes.

When a needle-liquid electric discharge occurs, an electric potential is formed in the liquid phase and requires more than several ten seconds to relax without ground^24^. Once potential is formed in medium including cell, the capacitors such as cell membrane and nuclei are charged depending on the voltage duration^38–40^. The voltage corresponds to the potential induced in a liquid medium by a pulsed discharge. A time difference between the first discharge and the subsequent discharge with a reverse polarity is 200 µs at maximum as shown in Fig. 3. Assuming that the relaxation time of the cell membrane potential in the HeLa cell is about the same millisecond as that of the nerve cell^32^, the maximum potential is contentiously applied to inside and outside the cell for 200 µs. Considering that such a potential difference leads the transient mechanical compressive force due to Maxwell stress, perforation may occur on the cell membrane. To evaluate this, the influence of potential stimulation on the cell membrane was studied. We added membrane-impermeable fluorescent dye to the medium before and after the stimulation, and microscopically observed the fluorescence-stained cells. This approach allows us to estimate whether the perforations formed in the membrane are temporary or stationary.

### Influence of potential stimulation on the cell membrane

**Figure 6:**
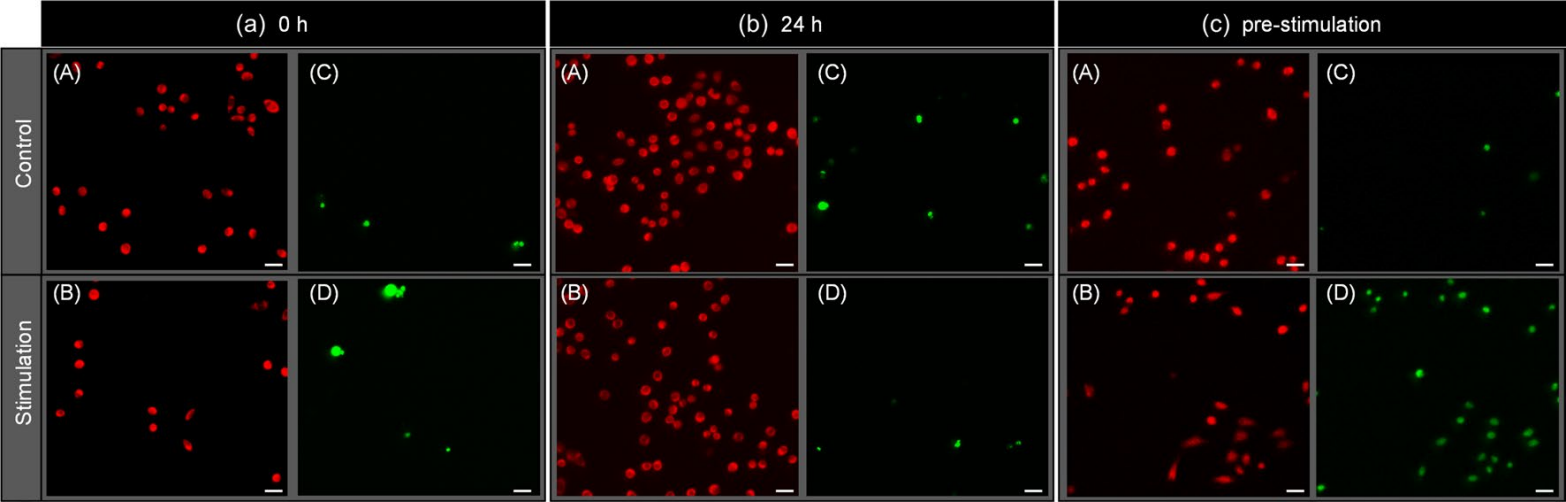
Result of fluorescent microscopic observation (scale bar: 40 µm). The fluorescent regents were added different time at (**a**) 0 h and (**b**) 24 h after potential stimulation. In (**c**) the fluorescence reagents were added before the stimulation. Red and green correspond to living cells and cells with pore, respectively.

**Figure 7:**
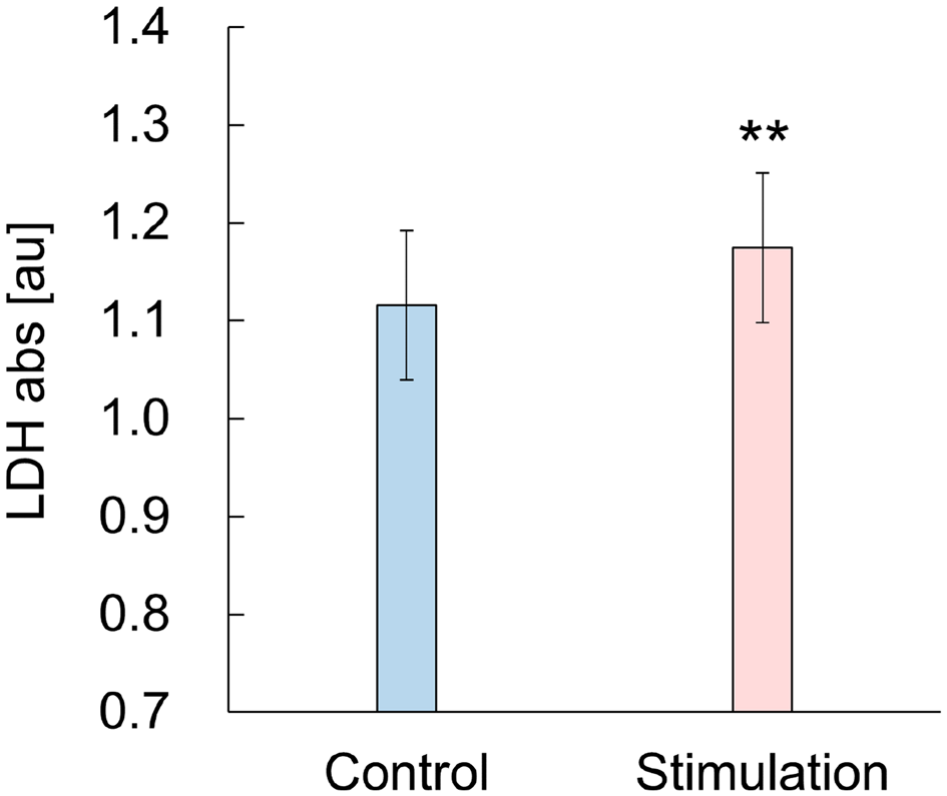
LDH level of each medium with and without potential stimulation for 30 min.

Fluorescence microscopy observations revealed that potential stimulation causes temporal pores on the membrane through which fluorescent reagent transports into the living cells. Figure 6 shows the result of the microscopic fluorescent observation for the cells with and without stimulation. To elucidate the biological effect of plasma-induced potential stimulation, fluorescent regents MitoRed and SYTOX-Green in DMSO were added at (a) 0 h and (b) 24 h after potential stimulation for 30 min and (c) before the stimulation. In Fig. 6a, cells were stained with MitoRed but not stained with SYTOX-Green. To quantitively evaluate the effect, the ratio of the number of cells simultaneously stained by both MitoRed and SYTOX-Green divided by the number of cells stained by MitoRed, *GR* ratio, were calculated. *GR* ratio enables us to assess the number of surviving cells with a non-lethal damage on the membrane. *GR* ratio was 0.097 in control group and 0.28 in stimulation group (Fig. 6a; Table 2). After 24 h (Fig. 6b), *GR* ratio was 0.12 in control group and 0.043 in stimulation group (Table 2). The number of MitoRed-stained cells with and without stimulation increased at almost the same, owing to proliferation. These results are consistent with Fig. 5. Conversely, many cells were stained by SYTOX-Green when added at before stimulation, even they were also stained by MitoRed (Fig. 6c). *GR* ratio was 0.16 in control group but 0.96 in stimulation group, which is significant high (Table 2). This result indicates that potential stimulation enables transport of SYTOX-Green into cells. It also should be noted that the cells with and without stimulation in Fig. 6a and b were not stained by SYTOX-Green when the reagent added at 0 h and 24 h later since stimulation. These results suggest that potential stimulation make temporal pores on the membrane of HeLa cells. Further experiment was conducted to evaluate the molecular transport out of the cell. LDH level of each medium after cell exclusion with and without potential stimulation for 30 min was measured. LDH is a soluble cytoplasmic enzyme that is present in almost all cells and is released to the medium when the membrane is damaged due to pores formation^41,42^. The control showed high LDH levels at 1.115 ± 0.008 as shown in Fig. 7. This is because phenol red and LDH that is originally contained in fetal bovine serum of the medium gives a positive bias to absorbance for colorimetry^43^. Nevertheless, potential stimulation shows slight but significant increase as 1.174 ± 0.045 than control (*p* = 0.021, n = 15). Above results show that potential stimulation induces molecular transport into and out of HeLa cell by temporal pores formation. This might be involved in the cell inactivation as shown in (Fig. 5). There are many reports on the formation of pores by atmospheric-pressure plasma irradiation to cells^44–48^. One of the mechanisms underlying pores formation is supplying plasma-induced radical species and other chemical species to the cell membrane, as Tero et al*.* have pointed out that lipid oxidation is involved in perforation^45^. This study revealed that potential simulation also has a key role for pores formation. It cannot be determined that all pores are reversible and thus there remains the possibility that the pores can be observed even after the treatment. Therefore, morphological change using SEM observations will be conducted in future.

**Table 2:** *GR* ratio obtained by dividing the number of cells simultaneously stained by both MitoRed and SYTOX-Green by the number of cells stained by MitoRed.

Fluorescent reagents were added at (a), (b), and (c)	Control	Stimulation
(a) 0 h after stimulation	0.097	0.28
(b) 24 h after stimulation	0.12	0.043
(c) Before stimulation	0.16	0.96

The result was obtained in three replicated.

### Possible mechanism underlying temporal poration by potential stimulation

**Figure 8:**
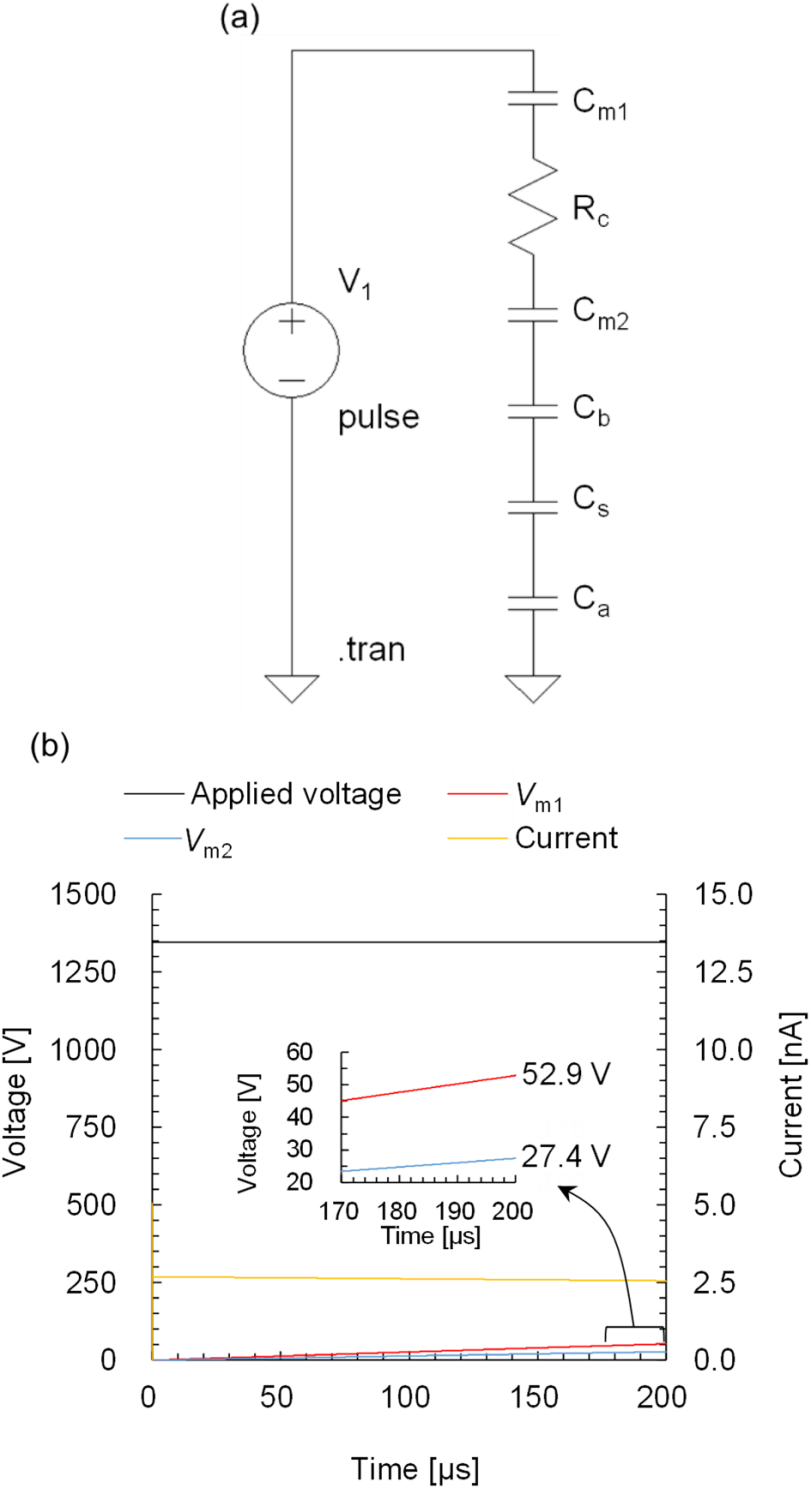
Numerical simulation using LTspice XVII. (**a**) Proposed equivalent circuit and (**b**) voltage and current.

Electric field across the membrane induced by the first discharge plasma (Fig. 3) was evaluated using LTspice XVII. Cell membrane potentials and continuous potential fluctuations between discharge current spikes were not addressed in this study. The discharge mode was the flashover^49^. As a result of the electron avalanche, the streamer shortens a circuit between the needle tip and the medium. The onset of the current spike and the start of the streamer propagation coincide in time. Based on a mean velocity of a streamer of 10^5^ m/s and the distance between the needle tip and the medium was 1.5 mm, the time required for shortening the circuit is calculated to be 15 ns^50^. The applied voltage hardly changes in this time. Thus, the potential that generates the current spike is imparted to the liquid at the maximum. This allows us to propose a simple equivalent circuit composed of an upper membrane *C*_m1_, a cytoplasm *R*_c_, a lower membrane *C*_m2_ of an adhered HeLa cell, bottom of polystyrene 96-well *C*_b_, blank space of 96-well plate (skirt) *C*_s_, and 200 mm space *C*_a_ as shown in Fig. 8a, where *C* is the capacitance and *R* is the resistance. These values ​​are calculated by Eqs. (3–4) as follows.

3 $$C=\frac{{\varepsilon }_{0}{\varepsilon }_{\mathrm{r}}}{d}$$

4 $$R=\frac{\rho l}{S}$$

5 $$\rho =\frac{1}{\sigma }$$

where ${\varepsilon }_{0}$ is electric constant as 8.854 × 10^−12^ F/m, ${\varepsilon }_{\mathrm{r}}$ is relative permittivity, *d* is distance of the capacitor (m), *ρ* is resistivity (Ωm), derived by (3), *l* is the length (m), *S* is the cross-section area of the resister (m^2^), and *σ* is the conductivity (mS/cm). *C*_m1_ and *C*_m2_ are both 9.929 × 10^−15^ F, *R*_c_ is 2.5 × 10^6^ Ω, *C*_b_ is 1.705 × 10^−20^ F, *C*_s_ is 6.542 × 10^−21^ F, *C*_a_ is 5.560 × 10^−23^ F based on the physical characteristics as follows. For HeLa cell, ${\varepsilon }_{\mathrm{r}}$ of cell membrane is 6.25, derived by averaging 5.7 for erythrocyte and 6.8 for lymphocyte, *d* is 7 nm, *σ* of cytoplasm is 3.2 mS/cm, and vertical *l* of cytoplasm of an adhered HeLa cell on the well bottom is 1 µm^51–53^. Since the horizontal radius of the adhered HeLa cell is 40 µm^54^, an equivalent circuit was constructed in the smallest unit by assuming that the other elements except voltage source were regarded as the same radius as well as the adhered HeLa cell. Voltage source was set as a pulse mode with 1.3 kV for 200 µs based on Fig. 3. For the 96-well plate, ${\varepsilon }_{\mathrm{r}}$ of polystyrene is 2.3^55^, *d* of bottom is 1.5 mm according to the product manufacturer. ${\varepsilon }_{\mathrm{r}}$ of air is 1.0. Figure 8b shows the simulation result. An applied voltage shows the potential of the medium with cell. *V*_m1_ and *V*_m2_ show the voltage across the upper and the lower membrane, respectively. The voltage applied to the membrane is divided depending on each capacitance. As shown in Fig. 8b as the current charges the capacitors, *V*_m1_ and *V*_m2_ gradually increase and reach 53 V and 27 V at 200 µs. The electric field was calculated as 7.6 × 10^3^ kV/mm for the upper membrane and 3.9 × 10^3^ kV/mm for the lower membrane. It is obvious that the electric field intensity decreases with a comprehensive equivalent circuit including all the intracellular components and more advanced components such as a membrane with channels and transporters. It should be noted that the simulation result also shows that a larger electric field is applied to the upper cell membrane, comparing to the lower cell membrane, *i.e.,* perforation may occur in the upper cell membrane. Deng et al*.* applied 6.0 kV of pulsed voltage between parallel plates inserted into a cell suspension at a distance of 1 cm and observed cell morphology after 15 min^38^. The cell morphology did not change as pulse width at 10 µs. In contrast, the cell membrane partially collapsed as that at 100 µs^38^. For this study, the electric field is maintained up to 200 µs. It was experimentally and theoretically shown that the potential induced in the liquid phase during plasma irradiation has a reversible perforation effect on the HeLa cell membrane. Plasma irradiation could be used to efficiently transport the generated RONS and other target molecules into cells.

## Conclusions

In this study, we investigated the biological effects of potential formation in the culture medium and cytoplasm induced by plasma irradiation on cells using cell activity, microscopic fluorescence observations, and a numerical simulation. A gas–liquid interfacial discharge plasma was generated by a bipolar pulse voltage, and only the potential fluctuation stimulus was given to the medium in which the cells were present. 30 min of electrical potential stimulation reduced the number of viable cells by about 85%. However, the cell proliferation rate after 24 h incubation was 2.1, which was almost the same as that of cells without stimulation. These results indicate that cells that remain active under potential stimulation can normally proliferate. Fluorescence microscopy has shown that fluorescent molecules that are impermeable to no-damaged cell membranes are transported into cells by potential stimulation. This was not observed when the fluorescent molecule is added after potential stimulation. Consequently, potential stimulation induces temporal perforation in the cell membrane. LDH measurement in the supernatant supports this result and suggests that potential stimulation can transport molecules in and out of cells. The numerical simulation on a transient analysis based on the physical properties of previous research and the actual applied voltage in this study showed that a larger electric field is induced inside and outside the upper membrane of HeLa cells adhering to the bottom than the lower membrane. In summary, we concluded that the mechanism underlying the potential-stimulated intracellular and extracellular molecular transport is primary pore formation on the upper membrane of HeLa cells. This study is the first report on the molecular transport into and out of cells by the potential formed in the liquid phase when irradiating a living cells with plasma. The potential fluctuation stimulation associated with plasma irradiation is a highly efficient molecular transport technology that does not inhibit cell proliferation, so it can be expected to be applied to medical treatment and agriculture. Transporting molecules into cells while maintaining cell activity is continuously required in fields such as regenerative medicine. The potential formation caused by plasma generation is decisive in the contribution of plasma science to cell biology and the elucidation of the mechanism underlying a biological response induction by plasma irradiation.

## Methods

### HeLa cell preparation

The HeLa cells was provided from Cell Resource Center for Biomedical Research, Institute of Development, Aging and Cancer, Tohoku University. HeLa cells were cultured in a culture medium composed of a regular medium which consists of minimum essential medium (MEM; Sigma, M4655-500) with 10% fetal bovine serum (FBS; Invitrogen 10437077) and 2% penicillin–Streptomycin mixed solution (Penicillin 10,000 µg/mL; Nacalai Tesque 26253-84). The electric conductivity of the culture medium was 9.53 mS/cm. We adjusted cell suspensions at 6.0 × 10^4^–1.0 × 10^5^ cell/mL using and prepared 100 μL/well in 96-well plate (Iwaki, 3861-096). After 24 h of incubation at 37 °C with 5% CO_2_, the cells were used for subsequent evaluation.

### Potential stimulation

Figure 2 shows the experimental set-up. As shown in the figure, two conditions of cells were prepared in a 96-well plate made of insulating material in a clean bench to prevent bacterial contamination. One experienced exposure to potential stimulation and the other was placed in the grounded medium (control). The distance between a needle tip and a medium's surface was 1.5 mm. A pulsed voltage with an amplitude of ± 7.5 kV, a rise time of 8 μs, width of 9 μs and 5 kpps of pulse repetition rate was applied for 30 or 60 min to the needle electrode by a function generator (NF, WF1973) and a high voltage amplifier (Trek, PD05034). Large current was prevented by insertion of dielectric material between the medium under the needle electrode and the ground. The potential fluctuation of medium to which direct discharges were generated was transferred through a SUS 316 cylindrical wire to the medium including HeLa cells. The tip of the cylindrical wire was submerged to a depth of 1 mm. During the treatment, the 96-well plate was placed 200 mm away from the ground to prevent the plasma generation at the bottom.

### Hydrogen peroxide assay

The H_2_O_2_ concentration of the medium was measured by colorimetry using a MEM eagle (GIBCO, 51200-038), instead of the MEM with phenol red, pack test reagent (Kyoritsu, WAK-H_2_O_2_) and digital pack test-Multi SP (Kyoritsu, DPM-MT). The limit of detection (LOD) was 0.05 mg/L. The conductivity of the medium including MEM eagle was 9.36 mS/cm.

### Cell concentration

The survival cell concentration was measured by cell count reagent SF (Nakarai tesque; 10% in the regular medium) and a microplate reader (Thermo Scientific, Multiskan FC) with the product protocol. The reagent was added to the sample and analyzed after an incubation for 4 h. See Fig. S1 in supporting information for the standard curve used in this study.

### Fluorescence microscopy

Fluorescence microscopy was used to elucidate the biological effect of plasma-induced potential stimulation using fluorescent regents MitoRed (Dojindo, 344-08851) and SYTOX-Green (Thermo, S7020). MitoRed stains living cells and SYTOX-Green stains cells with pores on their membrane. Both fluorescent reagents were simultaneously used to count surviving cells with damage on the membrane. The fluorescence reagents were added at different times, 0 h and 24 h after potential stimulation, and before the stimulation. The fluorescence images of the HeLa cells were taken by an inverted fluorescence microscope (Carl Zeiss, Axio Observer D1) using a 96-well glass bottom plate (Iwaki, 5866-096).

### Cell membrane response to potential stimulation

The damage to the cell membrane by potential stimulation was assessed by measuring lactate dehydrogenase (LDH) enzyme activity released into the medium using LDH Cytotoxicity Detection Kit (Takara, MK401) and a microplate reader with the product protocol. After potential stimulation, 80 µL of the supernatant and 40 µL of LDH assay reagent were mixed and incubated for 30 min in a 96-well. The absorbance of the samples was measured at 492 nm. The reference wavelength was 620 nm.

### Numerical simulation

A numerical simulation of the proposed equivalent circuit of an experimental system including an adhered HeLa cell was performed using LTspice XVII^56^ to assess the voltage across the membrane as the exposure to potential stimulation. The physical characteristics of the electrical elements are shown in the section of results and discussions.

### Supplementary Information

Supplementary Information.

## Data Availability

The datasets used and/or analysed during the current study available from the corresponding author on reasonable request.
