# Supplementary material for: Influence of electric potential-induced by atmospheric pressure plasma on cell response
Source: Sci Rep. 2023 Sep 25;13:15960. doi: 10.1038/s41598-023-42976-4 (PMC10520067; doi:10.1038/s41598-023-42976-4)
Supplement: Supplementary file 1 — Supplementary Information. [file 41598_2023_42976_MOESM1_ESM.docx]

**Supporting information**

**Influence of Electric Potential-Induced by Atmospheric Pressure Plasma on Cell Response**

*Takamasa Okumura ^1^, Chia-Hsing Chang ^2^, Kazunori Koga ^3^, Masaharu Shiratani ^4^, **Takehiko Sato ^5^

^1^ Graduate School and Faculty of Information Science, and Electrical Engineering, Kyushu University

^2^ Department of Mechanical System Engineering, Tohoku University, Sendai, Japan

^3^ Faculty of Information Science and Electrical Engineering, Kyushu University, Fukuoka, Japan

^4^ Faculty of Agriculture, Kyushu University, Fukuoka, Japan

^5^ Institute of Fluid Science, Tohoku University, Sendai, Japan

**Co-corresponding Authors:**

*Takamasa Okumura

744 Motoka, Nishi-ku, Fukuoka city, Fukuoka 819-0395, Japan

E-mail: t.okumura@plasma.ed.kyushu-u.ac.jp

**Takehiko Sato

2-1-1 Katahira, Aoba-ku, Sendai city, Miyagi 980-8577

E-mail: sato@ifs.tohoku.ac.jp

Figure caption.

**Fig. S1.** Standard curve for cell concentration.

**Fig. S1.**

Standard curve was obtained by colorimetry^1^ using cell count reagent SF (Nakarai tesque; 10% in the regular medium) and a microplate reader (Thermo Scientific, Multiskan FC). The OD value with the concentration was obtained at absorption at 450 nm (standard curve equation: *y* = 6E-06 *x* - 0.007, R² = 0.9998). The measurement was performed according to the protocol.

**References**

1. Tominaga, H. *et al.* A water-soluble tetrazolium salt useful for colorimetric cell viability assay. *Anal. Commun.* **36**, 47–50 (1999).
